# Stigma Scale Adaptation and Validation for Measuring COVID-19 Stigma

**DOI:** 10.7759/cureus.38744

**Published:** 2023-05-08

**Authors:** Puja Pallavi, Ajay K Bakhla, Prawin K Akhouri, Ravi R Kisku, Rajni Bala

**Affiliations:** 1 Psychiatry, Rajendra Institute of Medical Sciences, Ranchi, IND; 2 Medicine, Dr. Kiran C. Patel Medical College & Research Institute, Bharuch, IND; 3 Psychology, Rajendra Institute of Medical Sciences, Ranchi, IND

**Keywords:** psychosocial problems, validity, adaptation, stigma scale, covid-19

## Abstract

Background: The current coronavirus disease 2019 (COVID-19) pandemic has been found to be associated with increased psychosocial problems such as depression, anxiety, stress, and stigma. Many health-related stigma instruments that have been developed are condition-specific; these should be adapted and validated for generic use, across different health conditions. This study was conducted to measure stigma, stress, anxiety, and depression using the COVID-19 Stigma Scale-Modified (CSS-M), a modified version of the HIV Stigma Scale, among the Indian population.

Methods: A weblink-based online survey was conducted using the adapted CSS-M, along with the Depression, Anxiety, and Stress Scale-21. Collected data were analyzed with correlation analysis, reliability analysis, exploratory factor analysis, and convergent and divergent validity.

Results: With a sample size of 375, the modified scale for COVID-19 stigma showed internal consistency and a good inter-item correlation (Cronbach’s alpha 0.821). Principal axis factoring with varimax rotation along with alternative parallel analysis established the two factorial structure and had valid composite reliability, discriminate validity, and partial convergent validity.

Conclusion: We found that COVID-19 Stigma Scale-Modified is a valid measure to assess COVID-19-related stigma. The scale was found to be internally consistent with a good inter-item correlation, composite reliability, valid discriminate validity, and partial convergent validity. Specific COVID-related validated scales for stigma should be developed in the future.

## Introduction

Stigma is defined as an attribute linking a person to a set of undesirable characteristics like labeling, stereotyping, and separation that may lead to prejudice and discrimination [1]. Many illnesses like mental illnesses, leprosy, HIV/AIDS, and tuberculosis are known for social stigma and their detrimental effects [2-4]. Discrimination, prejudice, and stigma make sick people reluctant to get medical help [5,6]. Infectious disease pandemics have had a historical relationship with stigma and prejudice [7]. The outbreak of coronavirus disease 2019 (COVID-19) has created a heavy burden of psychological problems, including stigma, among the general population [8]. Many health-related stigma instruments that have been developed are condition-specific. However, the use of health-related stigma scales should be validated, developed, or adapted for generic use, across different health conditions where possible [9]. Population-specific scales are not available for the Indian population related to the new onset COVID-19. Therefore, this study was planned to adapt the Short-Version HIV Stigma Scale (HSS) as the COVID-19 stigma scale modified for validating the measurement of COVID-19 stigma in an Indian setting.

## Materials and methods

This was a cross-sectional, observational study carried out in India. The authors assert that all procedures contributing to this work comply with the ethical standards of the relevant national and institutional committees on human experimentation and with the Helsinki Declaration of 1975, as revised in 2008. All procedures involving human subjects/patients were approved by Institutional Ethics Committee (IEC) at Rajendra Institute of Medical Sciences, Ranchi, Jharkhand, India (IEC reg. no. ECR/769/INST/JH/2015/RR-18). The sample population of the study was the general population of India. The study included adults above 18 years of age, who had internet access and who understood the English language, to be able respond to the Google survey form. We collected data anonymously, without collecting information that could identify the respondents. The period of data collection was May 27 to June 27, 2020.

We used a purposive sampling technique for data collection. We also received permission from the original author to use the shorter version of the HIV Stigma Scale by email. The original HIV Stigma Scale was modified by replacing HIV with COVID-19; all other items’ wordings and scoring were kept similar and used for validation. Data was collected by using an online survey platform through Google Forms as per Government’s instruction to minimize face-to-face contact. Potential respondents were invited through emails and WhatsApp by sharing the survey link. Once the user clicked on the link, they got full information and the purpose of the survey, followed by a mandatory consent statement to be agreed upon on the first page. After giving consent to participate in the study, they were eligible for the survey. No name or any other personal identity data was obtained. Then a set of several questions appeared sequentially, which the participants were to answer. There was a request included for a single response from every respondent. In accordance with the accepted norm, a good sample size was calculated as over 360 (30 times 12 items of the ‘shorter version of the HIV Stigma Scale, modified for COVID-19’) for exploratory factor analysis.

Sociodemographic datasheet

The first part of the study questionnaire collected sociodemographic information including age, gender, marital status, education, habitat, history of physical illness, family history of psychiatric illness, and effect of information overload on stigma.

Shorter version of the HIV Stigma Scale modified for COVID-19

We called our adapted version COVID-19 Stigma Scale-Modified (CSS-M). This scale was adapted from the 12-item shorter version of the HIV Stigma Scale [10]. It was modified by replacing HIV with COVID-19; all other items’ wordings and scoring were kept similar to the original HIV Stigma Scale. This tool comprised a 12-item questionnaire that measures the stigma, consisting of four stigma subscales, including personalized stigma, disclosure concern, concern with public attitude, and negative self-image. Each subscale has three items that were rated along a four-point Likert scale (strongly disagree, disagree, agree, strongly agree). Responses were calculated as subscale scores with a possible range of 3-12; higher scores reflected a higher level of perceived stigma. Cronbach’s alpha (α) values for the subscales were all >0.7.

Depression, Anxiety, Stress Scale-21 (DASS-21)

The third part of the survey was adopted from DASS-21 (developed by Lovibond and Lovibond in 1995) to measure stress, anxiety, and depression with a total of 21 items [11]. DASS-21 consists of three subscales: Depression, Anxiety, and Stress. Each subscale has seven questions, with a final score for each scale obtained by summing up the scores of the relevant questions. The scoring pattern was a four-point Likert type. The scale’s reliability was analyzed with Cronbach’s α, varying according to the subscales: for depression, α = 0.076; anxiety, α = 0.82; stress, α = 0.75.

Statistical analysis

The data collected using the online Google Form was downloaded from the survey platform in Excel sheet format (Microsoft, Redmond, WA). The collected data was statistically analyzed using Statistical Package for Social Sciences, version 16.0 (SPSS Inc., Chicago, IL). There were 48 incomplete responses that were excluded from the study. Data normality was checked using skewness and kurtosis. Baseline sociodemographic characteristics were collected as categorical variables, except the age of participants, and presented descriptively as percentage, frequency, mean, and standard deviation. For the 12-item CSS-M, univariate summary statistics, Pearson's item-total correlations, scale means, if item deleted, and Cronbach’s alpha coefficients were calculated. A Cronbach’s alpha value of 0.70 or greater was accepted as the internal consistency of each subscale.

Exploratory factor analysis was carried out to identify factor structure of all items of CSS-M. The Kaiser-Meyer-Olkin (KMO) test of sample adequacy and Bartlett's test of sphericity were also done to assess the appropriateness of conducting factor analysis. For retaining the number of components, three criteria were considered: Kaiser’s criteria of eigenvalues greater than unity, Cattell’s scree plot inspection for the point of inflection, and Horn’s parallel analysis using Keeling’s regression equation [12-15]. Principal component analysis was carried out to identify the factor structure of the modified 12-item CSS-M. Orthogonal (varimax) rotation was carried out along with Kaiser’s normalization to identify the best solution. A cutoff of 0.3 in factor loading was considered significant as per Stevens’ recommendation.

For calculating the average variance extracted (AVE), the item loading (λ) of each factor was squared (λ^2^), added together, and divided by the number of items: composite reliability = (sum of λ^2^)/(sum of λ^2^ + sum of ε), where ε = 1 - λ^2^. Further calculation of the square root of AVE is known as discriminant value, used to establish discriminant validity (DV).

## Results

Demographics

A sample size of 375 was achieved after excluding 48 incomplete or duplicate responses. The mean age of the sample was 31.89 (SD 8.33) years. Most of the participants were male (53.6%), residing in urban areas (77.3%) and educated till graduation (45.9%) and postgraduation (41.1%) levels. Other sociodemographic variables and their distribution and frequency are tabulated in Table 1.

**Table 1:** Sociodemographic characteristics DASS: Depression, Anxiety, Stress Scale

	Mean (standard deviation)	Min.	Max.
Age	31.89 ± 8.33	19	62
		n	%
Gender	Male	201	53.6
	Female	174	46.4
Marital status	Unmarried	195	52
	Married	180	48
Education	Postgraduation	154	41.1
	Graduation	172	45.9
	Intermediate	36	9.6
	Matriculation	02	0.5
	Other	11	2.9
Habitat	Urban	290	77.3
	Suburban	60	16
	Rural	25	6.7
History of any physical illness	No	343	91.5
	Yes	32	8.5
Family history of any psychiatric illness	No	348	92.8
	Yes	27	7.2
DASS score above cutoff	Depression	78	20.8
	Anxiety	82	21.87
	Stress	48	12.8

DASS-21 score results

There were 20.8%, 21.87%, and 12.8% of general people who had depression, anxiety, and stress, respectively, as per scorings of DASS-21 (Table 1).

CSS-M score results

The 12-item CSS-M was used as a Likert scale of 1-4 scoring, ranging from strongly disagree to strongly agree. The frequency of choices in the percentage of individual items has been tabulated in Table 3. A total of 56% were assertive responses for stigma as agreed or strongly agreed. The mean scores and SDs of individual items were also tabulated that ranged from 1.68 ± 0.71 to 3.18 ± 0.71. The mean of the total scale score was 28.67 (SD 5.76) and the median score was 29.

Reliability internal consistency

The Cronbach’s alpha for the full scale was 0.821 and Cronbach’s alpha if the item was deleted ranged from 0.794 to 0.819. The scale means, if an item was deleted, ranged from 25.49 to 26.99 and Pearson’s item-total correlation was found to be significant (p = .000) (Table 2).

**Table 2:** Univariate summary statistics for the 12-item CSS-M, Pearson's item-total correlations and Cronbach’s alpha CSS-M: COVID-19 Stigma Scale-Modified *Assertive response for stigma as agree or strongly agree was 56%.

CSS-M items	Response option frequencies (%)				Mean (SD)	Scale mean, if item deleted	Pearson’s item‑total correlation	Cronbach's alpha, if item deleted
	Strongly disagree	Disagree	Agree*	Strongly agree*				
1	4.8	9.1	56.8	29.3	3.11 ± 0.75	25.57	.383	.815
2	22.9	44.8	24.5	7.7	2.17 ± 0.87	26.51	.630	.794
3	30.1	46.7	18.7	4.5	1.98 ± 0.82	26.70	.596	.798
4	11.5	19.5	44.8	24.3	2.82 ± 0.93	25.86	.418	.814
5	45.9	42.1	8.8	3.2	1.69 ± 0.76	26.98	.387	.815
6	19.5	32.3	37.9	10.4	2.39 ± 0.92	26.29	.501	.806
7	12.3	22.4	48.0	17.3	2.70 ± 0.89	25.97	.571	.799
8	16.3	42.1	34.1	7.5	2.33 ± 0.83	26.35	.482	.807
9	4.3	4.5	59.7	31.5	3.18 ± 0.71	25.49	.469	.809
10	37.6	42.9	16.0	3.5	1.85 ± 0.81	26.82	.481	.807
11	10.9	20.0	50.1	18.9	2.77 ± 0.88	25.91	.355	.819
12	44.0	46.4	7.2	2.4	1.68 ± 0.71	26.99	.418	.812
	Stigma total score, mean (SD)				28.67 ± 5.76			.821

External validity

The correlation between the total scores of CSS-M and DASS-21 anxiety, depression, stress, and the total score was found to be positively correlated with significance (Pearson’s correlation coefficient = 0.392; 0.370; 0.424; 0.242, respectively with p = .000, for all four correlations), suggesting that these scales seem to assess similar constructs affirming convergent validity.

Factor analysis

The KMO measure of sampling adequacy was 0.808, which indicates adequate sample size for the factor analysis. Bartlett’s test of sphericity was significant (χ^2 ^= 1.26, df = 66, p = .000), indicating that factor analysis was appropriate. Exploratory factor analysis was performed to know the CSS-M factor structure. Principal component analysis showed four components with an initial eigenvalue greater than unity (4.12, 1.39, 1.09, and 1.03), with 34.33%, 11.58%, 9.11%, and 8.55% of the variance, respectively, making a total of 63.58% variance. But the scree plot showed a point of inflection after two components. Horn’s parallel analysis was also used using Keeling’s regression equation, which showed criterion values of 1.29, 1.23, 1.17, and 1.11, respectively, for four factors. The first two criterion values were found to be smaller but third and fourth values were greater than the eigenvalue. Considering scree plot analysis and Horn’s parallel analysis, a two-factor solution was retained (Table 3).

**Table 3:** Principal axis factoring of the 12-item scale (varimax rotation with Kaiser normalization showing factor loadings >0.3) CSS-M: COVID-19 Stigma Scale-Modified *The initial eigenvalue was smaller than the criterion value from parallel analysis, and hence was rejected and excluded from further analysis. **Average variance extracted of factors above 0.5 is considered the conventional threshold of convergent validity. ***Composite reliability above the cutoff of 0.7 indicates the reliability of the factor construct.

		Principle component analysis with varimax rotation (N=375)			
	Initial eigenvalue (criterion value from parallel analysis)	4.12 (1.29)	1.39 (1.23)	1.09 (1.17)*	1.03 (1.11)*
	Items of CSS-M	1	2	3	4
12	I feel I’m not as good a person as others because I have Corona	.796			
10	I feel guilty because I have Corona	.739			
3	I have lost friends by telling them I have Corona	.610	.351		
2	People I care about stopped calling after learning I have Corona	.594	.314		
11	People’s attitudes about Corona make me feel worse about myself	.582			
8	Most people believe a person who has Corona is dirty		.834		
7	People with Corona are treated like outcasts		.784		
9	Most people are uncomfortable around someone with Corona		.525		
1	Some people avoid touching me once they know I have Corona			.820	
4	Telling someone I have Corona is risky			.556	
5	I work hard to keep my Corona a secret				.833
6	I am very careful who I tell that I have Corona		.365		.719
	Percentage of variance – initial (rotated)	34.33 (19.98)	11.58 (16.87)		
	Average variance extracted	0.45**	0.53**		
	Average shared variance	0.66	0.71		
	Discriminate value	0.68	0.73		
	Composite reliability	0.80***	0.76***		

Factor structure

The first factor named “Anticipated self-stigma” had rotated eigenvalue 1.29 and explained 19.98% of the variance. It reflected five items (12, 10, 3, 2, and 11), with the highest loading (0.796) with item 12 (“I feel I’m not as good a person as others because I have Corona”), whereas the lowest loading (0.582) was with item 11 (“People’s attitudes about Corona make me feel worse about myself”) (Table 3).

Convergent validity and discriminate validity

The average shared variance (ASV) for factor I was found to be 0.66; for factor II, it was 0.71. The AVE was calculated for the first and second factors and it was found to be 0.45 and 0.53, respectively. The AVE of factors above 0.5 was considered the conventional threshold of convergent validity. The AVE implicates invalid convergent validity for the first factor but validates for the second factor. Further discriminant value was calculated by the square root of AVE and found to be 0.68 and 0.73, respectively, for the first and second factors. Both of these values are higher than the highest variable correlation of 0.65, which also indicates valid discriminant validity. Furthermore, the composite reliability value was calculated to be 0.80 and 0.76 for factors I and II, respectively; both of these are above the cutoff of 0.7 indicating the reliability of each factorial construct.

Finally, based on these analyses, a modified scale for COVID-19-related stigma with eight items was finalized excluding four unrelated items (Table 3). With this modified scale, we found a total mean score of 18.67 ± 4.19 with 41.6% of participants responding in agreement to experiencing stigma related to COVID-19. For the first factor consisting of five items, there was 30.72% assertive response, but for the three items of the second factor, there were 59.74% assertive responses (Table 4).

**Table 4:** Distribution percentage of measured psychosocial problems as measured by DASS-21 and the modified stigma scale (N=375) DASS-21: Depression, Anxiety, Stress Scale-21 Factor I, anticipated self-stigma; Factor II, perceived public stigma

DASS score above cutoff	Severity-wise %		Total %
Depression	Mild	7.7	20.8
	Moderate	8.5	
	Severe	1.6	
	Extremely severe	2.9	
Anxiety	Mild	5.1	21.87
	Moderate	8.0	
	Severe	3.5	
	Extremely severe	5.3	
Stress	Mild	4.3	12.8
	Moderate	5.1	
	Severe	1.9	
	Extremely severe	1.6	
Stigma ratings, assertive responses	Factor I	30.72	41.6
	Factor II	59.74	

## Discussion

The unprecedented psychosocial impact of the pandemic necessitated a scale for stigma associated with COVID. The earlier modified Berger HIV Stigma Scale has been used and validated among children with HIV, patients with hepatitis C virus infection, Ebola survivors and leprosy patients [16-19]. We attempted this study for the purpose of adapting HSS as a quicker, reliable, and validated scale for COVID-19.

There are many other factor analytic studies on persons with HIV using the original HSS with 40-item scales extracting four factors [10,17,20-23]. Few other studies also found three factors [24,25]. There is only one study among HIV patients that reported higher order, bi-dimensional structures, named perceived external stigma and internalized stigma [22]. We also found a two-factor solution by exploratory factor analysis; however, a three-factor solution was reported by a similar study for the stigma of COVID-19. This earlier study had been done with a relatively small sample size and without the use of parallel analysis [25].

The first factor of our study “anticipated self-stigma” included three items of “negative self-image” and two items of “personalized stigma”, but the convergent validity could not be attained. The second factor consisting exclusively of “concern about public attitude” exhibited valid convergent validity (Table 3). The original four factors of the shorter version of HSS that were personalized stigma, disclosure concerns, negative self-image and concern about public attitude are appropriate for the context of HIV [10]. COVID-19 is unique in many aspects including the rapidity, contagiousness, and pandemic extent. The exclusion of all three items of “disclosure concern” and the first item “Some people avoid touching me once they know I have Corona” appeared very rational. In contrast to HIV, touch avoidance and disclosure are essential elements of corona prevention and cannot be a part of stigma (Table 3).

Psychological problems

In our study, we found depression, anxiety, and stress in 20.8%, 21.87%, and 12.8% participants, respectively, as measured by DASS-21. This is in concordance with a recent systemic review and meta-analysis that concluded COVID-related depressive symptoms among 12.5%-47.1%, anxiety features among 4.2%-50.3%, and psychological distress among 28.4%-48.2% [26]. The higher psychological distress was found to be associated with the younger age and female sex.

Interitem reliability and correlation of CSS-M

In our study, the CSS-M for COVID-19 appeared to show consistently high Cronbach’s α ranging from 0.794 to 0.819, indicating good inter-item reliability, and the instrument’s internal consistency. This was found to be in accordance with most of the recent studies [16,18,21-24,26,27].

Furthermore, the Pearson item-total correlation was found to be significant in our study suggesting that all the items in the CSS-M were correlated among themselves, and its total score affirms its reliability. Additionally, the total scores of CSS-M and subscales of anxiety, depression, and stress of DASS-21 were also positively correlated, suggesting external validity with anxiety, depression, and stress. This is in accordance with most of the research review findings on this issue [8].

Methodological issues

Data collection by an online survey was the only option during the COVID-19 pandemic in view of safety and included advantages like low cost, less time, and convenience. However, it also poses some issues like maintaining a clearly defined population, response rate, and incomplete partial response [28]. We adhered to many of the methodological procedures of the Checklist for Reporting Results of Internet E-Surveys (CHERRIES) statement to optimize the quality of the online survey [29]. We managed an adequate sample size for psychometric evaluation and a good sample size for factor analysis with a sample size of 375.

Limitations and implications for future research

This study had several potential limitations. The study was conducted during a period of lockdown, which can have its own psychological impact. The sample was also limited to persons with knowledge of the English language and technologically able to participate in an online survey; this limited its generalizability to the wider population of India. We could not assess the illness-specific concurrent validity, due to the lack of validated COVID-19 stigma instruments, which may have affected the accuracy of the findings. We started this study with no specific stigma scale for COVID-19 available anywhere; however, later on a similar study was published [25]. We agree with the uniqueness of an epidemic for its infectiousness, social fear, isolation, and quarantine, but an ideal scale is yet to be developed. We conducted exploratory factor analysis to identify the factor structure of the modified CSS-M. However, confirmatory factor analysis is also required to assess the stability of its factor structure. Therefore, specific COVID-related validated scales for stigma should be developed in the future.

## Conclusions

In this weblink-based online survey study, we used the COVID-19 Stigma Scale-Modified to check the scale reliability and validity with correlation analysis, reliability analysis, exploratory factor analysis, and convergent and divergent validity among the Indian population. This study found that CSS-M is internally consistent with good inter-item correlation, composite reliability, valid discriminate validity, and partial convergent validity with the study finding of very high stigma as measured by this scale. This study also found a very high prevalence of depression, anxiety, and stress among the population in relation to COVID-19.
